# GALR2 and Y1R agonists intranasal infusion enhanced adult ventral hippocampal neurogenesis and antidepressant‐like effects involving BDNF actions

**DOI:** 10.1002/jcp.30944

**Published:** 2023-01-04

**Authors:** Jose Erik Alvarez‐Contino, Estela Díaz‐Sánchez, Marina Mirchandani‐Duque, Jose Andrés Sánchez‐Pérez, Miguel A. Barbancho, Alexander López‐Salas, Natalia García‐Casares, Kjell Fuxe, Dasiel O. Borroto‐Escuela, Manuel Narváez

**Affiliations:** ^1^ Laboratorio NeuronLab, Instituto de Investigación Biomédica de Málaga, Facultad de Medicina Universidad de Málaga Malaga Spain; ^2^ Grupo Hospitalario Vithas Vithas Málaga Málaga Spain; ^3^ Unit of Psychiatry, Instituto de Investigación Biomédica de Málaga Hospital Universitario Virgen de la Victoria Málaga Spain; ^4^ Department of Neuroscience Karolinska Institute Stockholm Sweden; ^5^ Department of Biomolecular Science, Section of Physiology University of Urbino Urbino Italy

**Keywords:** depression, galanin, hippocampus, neurogenesis, neuropeptide Y

## Abstract

Dysregulation of adult hippocampal neurogenesis is linked to major depressive disorder (MDD), with more than 300 million people diagnosed and worsened by the COVID‐19 pandemic. Accumulating evidence for neuropeptide Y (NPY) and galanin (GAL) interaction was shown in various limbic system regions at molecular‐, cellular‐, and behavioral‐specific levels. The purpose of the current work was to evaluate the proliferating role of GAL2 receptor (GALR2) and Y1R agonists interaction upon intranasal infusion in the ventral hippocampus. We studied their hippocampal proliferating actions using the proliferating cell nuclear antigen (PCNA) on neuroblasts or stem cells and the expression of the brain‐derived neurothrophic factor (BDNF). Moreover, we studied the formation of Y1R–GALR2 heteroreceptor complexes and analyzed morphological changes in hippocampal neuronal cells. Finally, the functional outcome of the NPY and GAL interaction on the ventral hippocampus was evaluated in the forced swimming test. We demonstrated that the intranasal infusion of GALR2 and the Y1R agonists promotes neuroblasts proliferation in the dentate gyrus of the ventral hippocampus and the induction of the neurotrophic factor BDNF. These effects were mediated by the increased formation of Y1R–GALR2 heteroreceptor complexes, which may mediate the neurites outgrowth observed on neuronal hippocampal cells. Importantly, BDNF action was found necessary for the antidepressant‐like effects after GALR2 and the Y1R agonists intranasal administration. Our data may suggest the translational development of new heterobivalent agonist pharmacophores acting on Y1R–GALR2 heterocomplexes in the ventral hippocampus for the novel therapy of MDD or depressive‐affecting diseases.

## Graphical abstract

GALR2 and Y1R agonists intranasal infusion increased the neuroblasts proliferation and expression of brain‐derived neurothrophic factor (BDNF) in the ventral hippocampus of rats. Moreover, a combination of both agonists induced antidepressant effects, by forced swimming test, exerting BDNF a crucial role in this action.

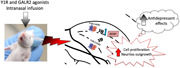

## 1 INTRODUCTION

Neurogenesis is a process within neuronal plasticity, the capacity of the brain to reorganize its structure, function, and connections in response to extrinsic or intrinsic stimuli (Baptista & Andrade, 2018). In this regard, new neurons are produced in the brain via a manner known as adult hippocampal neurogenesis (AHN). Among the neurogenic areas studied in the human brain, hippocampal formation emerges as crucial, exerting a noteworthy role in brain function (Spalding et al., 2013; Toda et al., 2019). In the dentate gyrus (DG) of the hippocampus is located the neurogenic niche, aimed to maintain AHN and implicated in many processes under normal physiological conditions during adulthood. Regarding AHN, there is an ongoing controversy among researchers. Some reports questioned AHN to be preserved throughout human life (Cipriani et al., 2018; Sorrells et al., 2018). On the other side, recent evidence support AHN until the ninth decade of life, being essential to keep related physiological functions (Boldrini et al., 2018; Moreno‐Jiménez et al., 2019; Terreros‐Roncal et al., 2022). Furthermore, a functional separation has been demonstrated in the hippocampus, where stress and modulation of emotional behavior are processed by the anterior portion in humans (ventral, in rodents) while cognitive functions and memory are related to the posterior part (dorsal, in rodents) (Fanselow & Dong, 2010; Kheirbek et al., 2013; Tanti & Belzung, 2013).

Intriguingly, dysregulation of AHN is related to several brain disorders, such as major depressive disorder (MDD), age‐dependent cognitive decline, Alzheimer's disease (AD), amyotrophic lateral sclerosis, Huntington's disease, Parkinson's disease, dementia with Lewy bodies, and frontotemporal dementia (I. B. Kim & Park, 2021; Martos et al., 2022; Moreno‐Jiménez et al., 2019; Terreros‐Roncal et al., 2022). MDD is one of the prominent mental health conditions in the world with more than 300 million people diagnosed. MDD is defined by a collection of behavioral, emotional, and cognitive symptoms, and confer a challenge for the medical community by increasing the risk of suicidality and death. Suicide is considered the worst outcome or consequence of MDD, over 700,000 human lives are lost every year (Elias et al., 2022). Moreover, during the COVID‐19 pandemic, multiple challenges have arisen, such as loneliness or financial hardship, producing about 34% prevalence of depression in general population, with 5%–15% suicidal ideation in that period (Giner et al., 2022).

The majority of current antidepressants target monoamines, with remarkable shortcomings such as adverse events and delayed onset of efficacy (Harmer et al., 2017). Notably, studies determine that approximately 50% of such patients fail to respond and about 65% of them fail to achieve remission, which classifies these patients with treatment‐resistant depression (TRD) (Chen, 2019). Recently, the discovery of ketamine as an effective antidepressant led to the authorization of a nasal spray form of esketamine (Hashimoto, 2020). Esketamine provides fast‐acting symptomatic relief in TRD patients, but the significant risks associated with esketamine limit its use for a broad patient population. Overall, nowadays MDD patients have no adequate treatment options, implicating that additional underlying mechanisms need to be considered to improve the efficacy of treatments. In recent decades, progress in the MDD field has been made possible in part through the use of rodent models (Planchez et al., 2019). Accordingly, boosting hippocampal neurogenesis in these patients emerges as a potential therapeutic approach (Colucci‐D'amato et al., 2020; Miller & Hen, 2015). Critical components of AHN are cell proliferation, neuronal differentiation, and survival, strictly controlled by multiple intrinsic or extrinsic epigenetic factors that could promote or suppress neurogenesis (Toda et al., 2019). These factors confer a pivotal role in understanding the importance of adult neurogenesis on physiological and pathological conditions (Kempermann et al., 2018). In this way, the neurogenic‐promoting effects of neurotransmitters/neuropeptides and neurotrophic factors are crucial regulators of neurogenic niche activities in health and disease (Kuhn, 2015).

Among them, neuropeptide Y (NPY) is one of the most abundant neuropeptides in the nervous system. NPY is a 36 amino acid polypeptide neurotransmitter highly conserved in mammals and involved in basic biological and pathophysiological functions, such as neuroendocrine secretions, mood regulation, feeding behavior, circadian rhythms, neuronal excitability, neuroplasticity, and memory (Kormos & Gaszner, 2013; Zaben & Gray, 2013). Regarding hippocampal neurogenesis, a proneurogenic role of NPY on hippocampal stem cells has been evidenced both in vitro (Howell et al., 2003, 2007) and in vivo (Decressac et al., 2011; Geloso et al., 2015). In preclinical models, it was found that reduced brain NPY in genetic and environmental models of MDD (Cohen et al., 2018; Jiménez‐Vasquez, Overstreet, et al., 2000; Jiménez Vasquez, Salmi et al., 2000). Reliable with the animal data, brain NPY is reduced in the postmortem brains of MDD patients who committed suicide (Kautz et al., 2017; Sah & Geracioti, 2013). With regard to treatment effects, all antidepressant procedures tested preclinically to date increase brain NPY (Bjørnebekk et al., 2006; Husum et al., 2000). In line with these findings, transgenic rats overexpressing hippocampal NPY show decreased depression‐like behaviors (Thorsell et al., 2000). Furthermore, recently demonstrated that intranasal NPY and the NPY Y1 receptor (Y1R) agonist administration had antidepressant effects in rodents (Nahvi et al., 2021; Serova et al., 2017) and in MDD patients (Mathé et al., 2020). In this respect, the Y1R has been proposed as a critical target by mediating dentate neurogenesis and antidepressant effects (Rana et al., 2022).

Brain‐derived neurotrophic factor (BDNF) is a pivotal molecule involved in the neuroprotective effects of antidepressants by regulating different neurogenic processes in the hippocampus (Castrén & Kojima, 2017; Colucci‐D'amato et al., 2020; Miranda et al., 2019). Moreover, a reduction in BDNF was reported in the hippocampus of postmortem brain tissues of MDD and suicide victims (Dwivedi, 2012; Pandey et al., 2008) while injection of BDNF in the hippocampus reduces depression‐like behavior in rodents (Hoshaw et al., 2005). Besides, BDNF was significantly increased 24 h following treatment with NPY in the trimethyltin‐induced model of hippocampal neurodegeneration (Corvino et al., 2014).

Galanin (GAL) is a neuropeptide broadly distributed in the central nervous system contributing to numerous physiological effects (Katsetos et al., 2001). GAL effects on hippocampal precursor cells were elucidated by using the GAL2 receptor (GALR2)/GAL 3 receptor agonist, GAL 2–11, with proliferative and trophic actions in vitro (Abbosh et al., 2011). Accordingly, GALR2 is extensively expressed in the rat brain while the hippocampus presents high expression for GALR2 (Branchek et al., 2000). Concerning depression, all GALRs are involved in depression‐related behaviors with different roles via the modulation of neuroendocrine and monoaminergic systems (Fuxe et al., 2012; Wrenn & Holmes, 2006). Thus, intracerebroventricular (icv) infusion of GAL, and stimulation of GALR1 and GALR3 antagonists results in depressive‐like effects (Barr et al., 2006; Kuteeva et al., 2007, 2008). Conversely, GALR2/GALR3 activation after icv infusion induced antidepressant‐like effects in rats, and the increased GALR2 expression in the ventral hippocampus was related to antidepressant effects (Kuteeva et al., 2008; Luo et al., 2019). Moreover, GalR2‐knockout mice displayed depression‐like behaviors (Lu et al., 2008). Recently, the intranasal infusion of a spexin‐based GALR2 agonist‐induced antidepressant‐like effects in mice (Yun et al., 2019).

We have demonstrated NPY and interactions in different limbic system regions at molecular‐, cellular‐, and behavioral‐specific levels (Mirchandani‐Duque et al., 2022; Narváez et al., 2016, 2018, 2015). Previous work described a facilitatory interaction between NPY and GAL through the Y1R–GALR2 heteroreceptor complexes formation. We showed that Y1R–GALR2 heteroreceptor complexes on the ventral hippocampus might mediate the proliferative and antidepressant‐like actions following GAL and Y1R agonist icv infusion (Borroto‐Escuela et al., 2021). However, the translational impact of this invasive route is limited by the potential side effects and adverse events. In this regard, we performed intranasal delivery of combined GALR2 and Y1R agonists, improving spatial memory performance related to increased cell proliferation in the DG of the dorsal hippocampus (Borroto‐Escuela et al., 2022).

The purpose of the current work was to test the hypothesis that GALR2 and Y1R agonists intranasal infusion stimulates adult ventral hippocampal neurogenesis and exerts an antidepressant‐like effect. Thus, following GALR2 and Y1R agonists intranasal administration, we analyzed the ventral hippocampal activation and their proliferating actions through c‐Fos expression and proliferating cell nuclear antigen (PCNA). Moreover, we studied the specific cell subpopulation that is stimulated to proliferate with double immunolabeling To examine the associated cellular mechanism we assessed the expression of the BDNF on the ventral hippocampal DG. Moreover, we studied the formation of Y1R–GALR2 heteroreceptor complexes with in situ proximity ligation assay (PLA) and analyzed morphological changes on hippocampal neuronal cells. Finally, the functional outcome of the NPY and GAL interaction on the ventral hippocampus was evaluated in the forced swimming test (FST), and the role of BDNF in this action. We present consistent data employing the noninvasive intranasal delivery that takes advantage of a direct nose‐to‐brain transport of therapeutics, offering an alternative to icv injection.

## 2 MATERIALS AND METHODS

### 2.1 Animals

Male Sprague–Dawley rats from CRIFFA (200–250 g; 6–8 weeks) had free access to food pellets and tap water. They were maintained under the standard 12 h dark/light cycle, with controlled temperature (22 ± 2°C) and relative humidity (55%–60%). All procedures concerned with housing, maintenance, and experimental treatment of the rats were approved by the Local Animal Ethics, Care, and Use Committee for the University of Málaga, Spain. Guidelines for animal experiments were carried out following EU Directive 2010/63/EU and Spanish Directive (Real Decretory 53/2013) recommendations.

### 2.2 Drugs used

Solutions were freshly prepared in distilled water. GAL receptor 2 agonists (M1145), Y1R receptor agonist [Leu^31^, Pro^34^]NPY, GALR2 antagonist M871 were purchased from Tocris Bioscience and TrkB antagonist (ANA‐12, 5.06304) from Sigma‐Aldrich. Detailed descriptions are available in Supporting Information: Material on intranasal infusion of solutions.

### 2.3 Assessment of ventral hippocampus activation after intranasal infusion

Animals were randomly allocated into five experimental groups: (1) Control: distilled water; (2) M1145‐ treated group (132 µg); (3) Y1R agonist‐treated group receiving the Y1R agonist [Leu^31^–Pro^34^]NPY (132 µg); (4) M1145 + Y1R: group administered with both substances; (5) M1145 + Y1R + M871: group treated with M1145, [Leu^31^–Pro^34^]NPY and the GALR2 antagonist (M871; 132 µg) (*N* = 4 in each group). The doses indicated are based on previously published protocols (Borroto‐Escuela et al., 2022; Serova et al., 2017).

Twenty‐four hours after the intranasal administration, rats were deeply anesthetized with pentobarbital (mebumal, 100 mg/kg, ip) and transcardially perfused with 4% paraformaldehyde (wt/vol; Sigma‐Aldrich). Using a Cryostat (HM550; Microm International), the brains were coronally sliced (30 μm thick) through the ventral hippocampus (anterior in primates) (−5.20 to −6.72 Bregma; Paxinos & Watson, 2006).

We used the c‐Fos immunohistochemistry, as an indirect marker of neuronal activation. Free‐floating sections were incubated for antigenical retrieval at 65°C for 90 min in saline sodium citrate buffer (pH 6; 10 nM sodium citrate). After this procedure to remove endogenous peroxidases, the slices were treated for 30 min in 0.6% H_2_O_2_. Then, slices were incubated at 4°C overnight with a primary antibody mouse anti‐c‐Fos protein (1:800 sc‐271243; Santa Cruz Biotechnology) in 2.5% donkey serum. After several washes with phosphate‐buffered saline (PBS), the slices were incubated with a secondary antibody for 90 min (biotinylated anti‐mouse immunoglobulin G [IgG], 1:300, B8520; Sigma‐Aldrich). Then, ExtrAvidin peroxidase (1:100; Sigma‐Aldrich) was used to amplify the specific signal for 1 h at room temperature (RT) in darkness. Detection was performed with 0.05% diaminobenzidine (DAB; Sigma‐Aldrich) and 0.03% H_2_O_2_ in PBS. After several washes, slices were mounted on gelatin‐coated slides, dehydrated in graded alcohols, and cover‐slipped with DePeX mounting medium (Merck Life Science S.L.U.). C‐Fos‐labeled cells were studied using the optical fractionator method in unbiased stereological microscopy (Olympus BX51 Microscope), as previously described (Borroto‐Escuela et al., 2022; Mirchandani‐Duque et al., 2022; Narváez et al., 2018) (see Supporting Information: Materials for details).

### 2.4 Evaluation of ventral hippocampal cell proliferation and BDNF induction

Different free‐floating sections were incubated for antigenical retrieval at 65°C for 90 min in saline sodium citrate buffer (pH 6; 10 nM sodium citrate). After this procedure to remove endogenous peroxidases, the slices were treated for 30 min in 0.6% H_2_O_2_. Then, a set of slices were incubated at RT overnight with a primary antibody mouse anti‐PCNA (1:1500, P8825; Sigma‐Aldrich) or a different one with mouse anti‐BDNF (Abcam; ab205067, 1:500) in 2.5% donkey serum. After several washes with PBS, the slices were incubated with a secondary antibody for 90 min (biotinylated anti‐mouse IgG, 1:200, B8520; Sigma‐Aldrich). Then, ExtrAvidin peroxidase (1:100; Sigma‐Aldrich) was used to amplify the specific signal for 1 h at RT in darkness. Detection was performed with 0.05% DAB (Sigma‐Aldrich) and 0.03% H_2_O_2_ in PBS. After several washes, slices were mounted on gelatin‐coated slides, dehydrated in graded alcohols, and cover‐slipped with DePeX mounting medium (Merck Life Science S.L.U.). PCNA and BDNF‐labeled cells were studied using the optical fractionator method in unbiased stereological microscopy (Olympus BX51 Microscope; Olympus), as described above.

To study the specific cell subpopulation, we performed double immunolabeling. Procedures for double immunohistochemistry were previously described (Narváez et al., 2016). The PCNA immunostaining was performed as described above revealed with DAB plus 0.03% nickel (Sigma‐Aldrich) to get a black‐purple reaction. For the second primary antibody, rabbit anti‐doublecortin (DCX; Abcam; ab18723, 1:2000) or rabbit anti‐glial fibrillary acidic protein (GFAP; Abcam; ab7260, 1:1500), the chromogen used was DAB to get a brownish reaction.

### 2.5 Hippocampal cell culture and conditions

Rat primary hippocampal neuronal cells were purchased from QBM Cell Science and cultured in Neurobasal medium supplemented with 10% fetal bovine serum, 2 mM GlutaMAX‐1, 1 mM sodium pyruvate, 100 U/ml penicillin G, and 100 μg/ml streptomycin and 2% B‐27 supplement at 37°C in a humidified 10% CO_2_ environment according to manufacturer's instructions. Half part of the medium was changed every 3 days. The cells were grown under the above conditions (control condition) for 7 days. Cultured hippocampal neurons were grown and treated for 24 h under specific pharmacologic conditions. Treated hippocampal cells were divided into experimental groups: (1) Control group; (2) M1145‐treated group (100 nM); (3) Y1R agonist‐treated group receiving an NPYY1R agonist [Leu^31^, Pro^34^]NPY (100 nM); (4) GAL + Y1R: Group administered with both substances; and (5) GAL + Y1 + M871: Group injected with GAL, [Leu^31^, Pro^34^]NPY and the GALR2 antagonist (M871; 1 μM). Cells were grown on poly‐d‐lysine‐coated glass coverslips and fixed with 4% formaldehyde solution for 20 min followed by two washes with PBS containing 20 mM glycine to quench the aldehyde groups.

### 2.6 In situ PLA and analysis of neurite length

To study the GALR2‐Y1R heteroreceptor complexes, the in situ PLA (in situ PLA) was performed as described previously (Borroto‐Escuela et al., 2021; Narváez et al., 2021). After permeabilization with PBS containing 0.2% Triton X‐100 for 5 min, cells were treated with PBS containing 1% bovine serum albumin. The hippocampal cells were then incubated with the primary antibodies diluted in a suitable concentration in the blocking solution at 4°C overnight. Then, cells were washed twice, and the proximity probe mixture (Duolink PLA probe anti‐goat MINUS and Duolink PLA probe anti‐rabbit PLUS; Sigma‐Aldrich) was applied to the samples and incubated for 1 h at 37°C in a humidity chamber. The unbound proximity probes were removed by washing the slides twice, 5 min each time, with blocking solution at room temperature under gentle agitation and the sections were incubated with the hybridization‐ligation solution (bovine serum albumin, 250 g/ml), T4 DNA ligase (final concentration of 0.05 U/μl), 0.05% Tween‐20, 250 mM NaCl, 1 mM ATP, and the circularization or connector oligonucleotides (125–250 nM), and incubated in a humidity chamber at 37°C for 30 min. The excess of connector oligonucleotides was removed by washing twice, for 5 min each, with the washing buffer A (Sigma‐Aldrich; Duolink Buffer A; 8.8 g NaCl, 1.2 g Tris Base, 0.5 ml Tween 20 dissolved in 800 ml high purity water, pH to 7.4) at room temperature under gentle agitation and the rolling circle amplification mixture (Duolink amplification red, DUO82011; Sigma‐Aldrich) was added to the cells and incubated in a humidity chamber at 37°C for 100 min. Then, the cells were incubated with the detection solution in a humidity chamber at 37°C for 30 min. In the last step, the cells were washed twice in the dark, for 10 min each, with the washing buffer B (Sigma‐Aldrich; Duolink Buffer B (5.84 g NaCl, 4.24 g Tris Base, 26.0 g Tris‐HCl dissolved in 500 ml high purity water, pH 7.5) at room temperature under gentle agitation. The coverslips were put on a microscope slide and a drop of appropriate mounting medium (e.g., Duolink Mounting Medium; Sigma‐Aldrich) was applied and sealed with nail polish. The slides were protected against light and stored for several days at −20°C before confocal microscope analysis. The in situ PLA experiments were performed using the following primary antibodies: rabbit anti GALR2 (Alomone Lab, 1:100) and goat anti NPYY1R (sc‐21992; Santa Cruz Biotechnology Inc.; 1:200). Furthermore, cells were labeled with Neuro‐Chrom Pan Neuronal Marker primary antibody (ABN2300, 1:100, Sigma‐Aldrich; Merck Life Science S.L.U.) for 1 h, extensively washed, and stained with the green fluorescence secondary antibody goat anti‐rabbit DyLight 488 (Jackson Laboratories InmunoResearch; 1:100). Cell nuclei were counterstained with 4′,6‐diamidino‐2‐phenylindole (blue) contained in the mounting medium. The negative control consists of the omission of the species‐specific primary antibody corresponding to the GALR2 in the presence of the two PLA probes. As a positive control of the PLA assay, a parallel analysis of the 5‐HTR1A–5HTR2A isoreceptor complexes has been performed as previously documented (Borroto‐Escuela et al., 2017). Acquisition of microscopy images, in situ PLA data analysis, and morphometric quantifications were performed as previously described (Narváez et al., 2020).

### 2.7 Assessment of depression‐like behavior in rats

Depression‐like behavior was assessed in the FST, originally proposed as a model of stress‐induced depression‐like behavior (Porsolt et al., 1977). FST is broadly used for the early screening of novel molecules with putative antidepressant‐like (AD) effects since immobility is commonly qualified as “despair” and is considered to reflect depression‐like states. Remarkably, the immobility response in the FST can be prevented by various types of AD treatments, including tricyclic antidepressants, monoamine oxidase inhibitors, selective serotonin reuptake inhibitors, and NA reuptake inhibitors (Planchez et al., 2019).

Behavioral experiments were performed between 09:00 and 14:00 h. Animals were adapted to handling and were taken into the experimental room (80–90 lux) for at least 1 h to reach habituation and assigned randomly to the experimental groups. Peptides were freshly prepared and intranasal treatments were administered 24 h before the test phase (20 μl total volume). Doses for GAL receptor 2 agonists (M1145), Y1R receptor agonist [Leu^31^, Pro^34^]NPY, GALR2 antagonist M871, and ANA‐12 were chosen based on previously published protocols (Borroto‐Escuela et al., 2022; Ribeiro et al., 2020; Serova et al., 2017). We performed dose–response curves to determine effective doses. A separate group of rats was randomly allocated into six experimental groups: (1) Control: distilled water; (2) M1145‐treated group (132 µg); (3) Y1R agonist‐treated group receiving the Y1R agonist [Leu^31^–Pro^34^]NPY (132 µg); (4) M1145 + Y1R: group administered with both substances; (5) M1145 + Y1R + M871: group treated with M1145, [Leu^31^–Pro^34^]NPY and the GALR2 antagonist (M871; 132 µg); (6) M1145 + Y1R + ANA‐12: group treated with M1145 [Leu^31^–Pro^34^]NPY and the TrkB antagonist (ANA‐12, 5.06304; Sigma‐Aldrich; 0.5 mg/kg, ip) (*N* = 6 in each group).

Swimming sessions were conducted by placing individually the rats in cylinders containing water (25 ± 0.2°C), 30 cm deep. Two sessions were conducted: an initial 15 min pretest followed 48 h later by a 5‐min test. The water in the cylinders was changed after every trial. The FST was performed according to previously reported methods (Borroto‐Escuela et al., 2021; Koike & Chaki, 2014). The total duration of floating (immobility) and swimming periods were scored during the 5 min test and analyzed using the Raton Time 1.0 software (Fixma S.L.). Floating in the water without struggling and only making movements necessary to keep its head above the water was regarded as immobility. Swimming was scored when they actively swam around the cylinder. Following swimming sessions, the rats were removed from the tank, carefully dried in heated cages, and then returned to their home cages. Behavioral experiments were carried out by observers blinded to all experimental conditions.

### 2.8 Statistical analysis

Data are presented as mean ± SEM, and sample number (*n*) is indicated in figure legends. GraphPad PRISM 8.0 (GraphPad Software) was used to analyze all data. One‐way analysis of variance (ANOVA) followed by the Newman–Keuls comparison posttest was performed. For comparing two experimental conditions, Student's unpaired *t* test statistical analysis was achieved. Differences were considered significant at *p* < 0.05 (*p < 0.05; **p < 0.01; ***p < 0.001).

## 3 RESULTS

### 3.1 The ventral DG is activated under GALR2 and the Y1R agonists intranasal infusion

**Figure 1:**
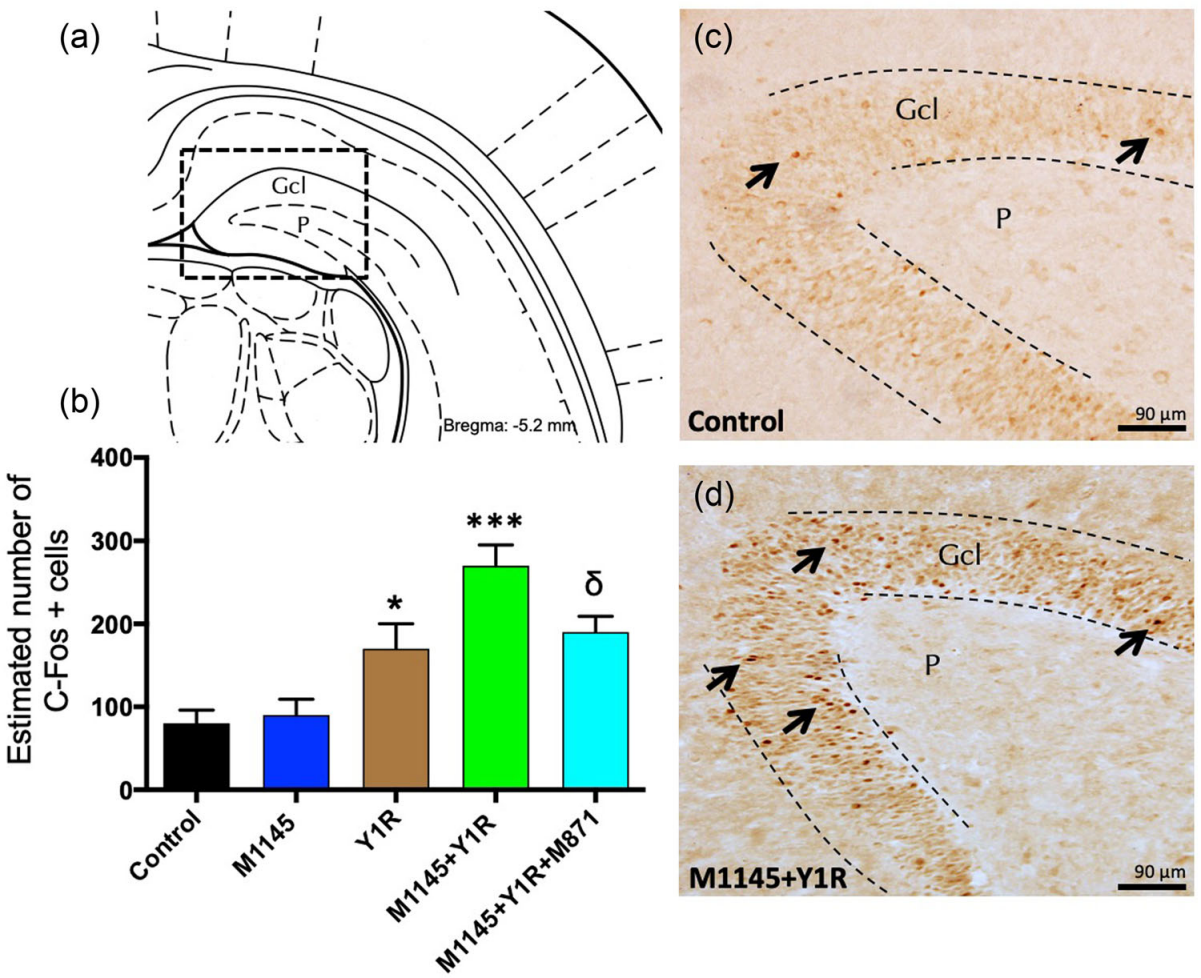
Ventral dentate gyrus is activated under galanin 2 receptor (GALR2) and the Y1R agonists intranasal delivery. Effects of the intranasal administration of GALR2 agonist (M1145) and Y1R receptor agonist, either alone or in combination with or without the GAL 2 receptor antagonist (M871) on c‐Fos expression in the granular layer of the ventral dentate gyrus. (a, d) The majority of the c‐Fos‐IR profiles were located in the granular cell layer (Gcl). It is also indicated by the polymorphic layer (P) of the dentate gyrus in the ventral hippocampus (Bregma: −5.6 mm; according to the Paxinos and Watson (2006) stereotaxic atlas). (b) Quantification of the total number of c‐Fos IR nuclei within the dentate gyrus of the ventral hippocampus. Data, expressed as mean ± SEM, show the differences between groups after administration of control, M1145, Y1R agonist [Leu^31^–Pro^34^]NPY, or the coadministration of both agonists with or without M871. *p < 0.05 versus control, M1145 and M1145 + Y1R; ^δ^ *p* < 0.05 versus M1145 + Y1R; ***p < 0.001 versus control and M1145 according to one‐way ANOVA followed by Newman–Keuls post hoc test (*n* = 4 in each group). Intergroup comparisons are indicated by the vertical lines from the horizontal line above bars. Intranasal coadministration of M1145 and Y1R agonist (d) increased the c‐Fos‐IR nuclei in Gcl in the dentate gyrus compared with the control group (c). Arrows indicate examples of c‐Fos‐IR nuclei. Dashed lines outline the Gcl of the dentate gyrus. ANOVA, analysis of variance; Control, distilled water; IR, immunoreactivity; M1145, galanin 2 receptor agonist 132 µg; M1145 + Y1R, coadministration of M1145 and Y1R; M1145 + Y1R + M871, coadministration of M1145, Y1R, and GALR2 antagonist M871 132 µg; Y1R, Y1R receptor agonist [Leu^31^–Pro^34^]NPY 132 µg.

To demonstrate the intranasal delivery of GALR2 and the Y1R agonists across the blood–brain barrier we assess the c‐fos induction, a marker of neuronal activation, on the ventral DG. The intranasal administration of the Y1R agonist induced a significant increase in the number of c‐fos‐IR profiles in the granular region of the ventral DG (one‐way ANOVA, *F*4, 15 = 12.04, *p* < 0.001, Newman–Keuls post hoc test: *p* < 0.05) (Figure 1a,b) compared with the control group. Conversely, the intranasal administration of the GalR2 agonist M1145 alone lacked effects on the numbers of c‐fos positive cells (Figure 1b) compared with the control group (Figure 1b,c)

Furthermore, the intranasal infusion of M114545 and Y1R agonist significantly increased the number of c‐Fos‐IR profiles in the granular region of the ventral DG compared with the M1145 and the control groups (Newman–Keuls post hoc test: *p* < 0.001) and with the Y1R agonist alone group (Newman–Keuls post hoc test: *p* < 0.05) (Figure 1b,d). The cotreatment with the GALR2 antagonist M871 specifically blocked this M1145 and Y1R agonists coadministration effects in the ventral DG (Newman–Keuls post hoc test: *p* < 0.05) (Figure 1b), indicating the participation of GALR2 in the Y1R‐M1145 agonists interaction to stimulate c‐fos induction.

### 3.2 GALR2 and the Y1R agonists intranasally administered increased neuroblasts cell proliferation in the ventral hippocampus

**Figure 2:**
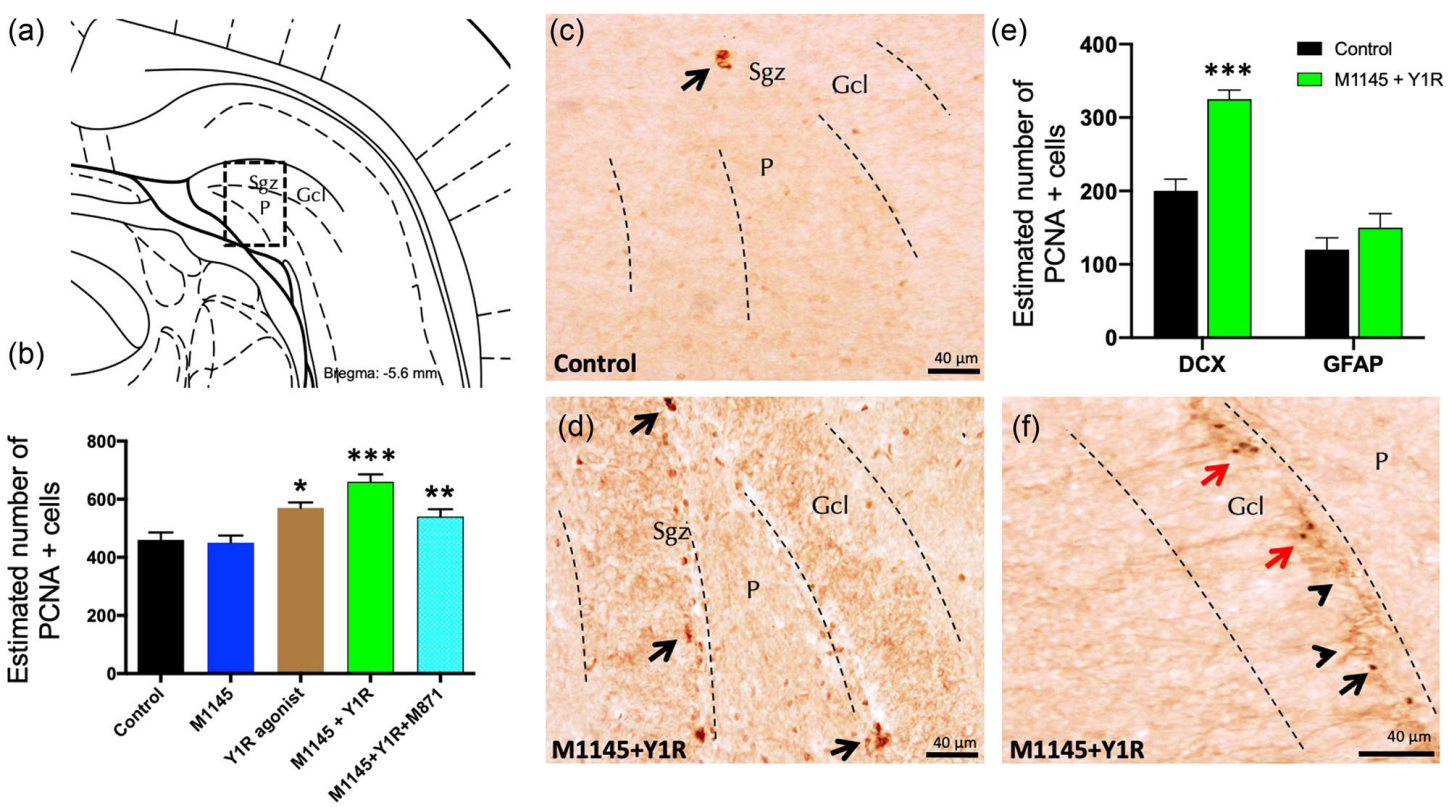
Intranasal (in) coadministration of galanin receptor 2 and Y1R agonists increases cell proliferation in the ventral dentate gyrus of adult rats. Proliferating cell nuclear antigen immunolabeling (PCNA) immunolabeling (PCNA+) in the dentate gyrus of the ventral hippocampus, after the in administration of galanin 2 receptor agonist (M1145) and Y1R receptor agonist, either alone or in combination with or without the GAL 2 receptor antagonist (M871). (a, d) The majority of the PCNA‐positive cells were located in the subgranular zone (Sgz) of the dentate gyrus at the border between the granular cell layer (Gcl) and the polymorphic layer (P) of the dentate gyrus in the ventral hippocampus. They appeared as clusters of 3–4 cells. (Bregma: −5.6 mm; according to the Paxinos and Watson (2006) stereotaxic atlas). (b) Quantification of total PCNA‐IR cells in the dentate gyrus of the ventral hippocampus. Data represent mean ± SEM to show the differences between groups after administration of control, M1145, Y1R agonist [Leu^31^–Pro^34^]NPY, or the coadministration of both agonists with or without M871. *p < 0.05 versus control, M1145 and M1145 + Y1R; **p < 0.01 versus M1145 + Y1R; ***p < 0.001 versus control and M1145 according to one‐way ANOVA followed by Newman–Keuls post hoc test. Intergroup comparisons are indicated by the vertical lines from the horizontal line above bars. *N* = 4 in each group. M1145 and Y1R agonist in coadministration (d) increased the PCNA immunolabeling in Sgz in the dentate gyrus compared with the control group (c). Arrows indicate examples of clusters of PCNA‐positive nerve cells. Dashed lines outline the Gcl of the dentate gyrus. (e) Quantification of PCNA‐IR cells double‐labeled with DCX or GFAP in either control or M1145 + Y1R‐administered rats revealed that Y1R–GALR2 specifically acts onto the neuroblasts. Data represent mean ± SEM. ***p < 0.001 versus control according to Student's unpaired *t* test statistical analysis. (f) Representative photomicrograph illustrating DCX+/PCNA+ cells (as indicated by red arrows), DCX−/PCNA+ cells (as indicated by red arrows) and DCX+/PCNA− cells (as indicated by arrowheads) in the M1145 and Y1R agonist group. ANOVA, analysis of variance; Control, distilled water; DCX, doublecortin; GFAP, glial fibrillary acidic protein; IR, immunoreactivity; M1145, galanin 2 receptor agonist 132 µg; M1145 + Y1R, coadministration of M1145 and Y1R; M1145 + Y1R + M871, coadministration of M1145, Y1R, and GALR2 antagonist M871 132 µg; Y1R, Y1R receptor agonist [Leu^31^–Pro^34^]NPY 132 µg.

We evaluated the impact of GALR2 agonist M1145 and the Y1R agonist intranasally coinjected on adult ventral hippocampal cell proliferation by using the PCNA. Intranasal M1145 and the Y1R agonist delivery significantly increased cell proliferation, as demonstrated by the number of PCNA‐IR cells, specifically in the subgranular zone (Sgz) of the DG compared to control (one‐way ANOVA, *F*4, 15 = 12.38, *p* < 0.001, Newman–Keuls post hoc test: *p* < 0.001) (Figure 2a,b,d), M1145 (Newman–Keuls post hoc test: *p* < 0.001) and Y1R agonist groups (Newman–Keuls post hoc test: *p* < 0.05) (Figure 2a,b,d). The addition of GALR2 antagonist M871 completely blocked the M1145 and the Y1R agonist effects in the DG (Newman–Keuls post hoc test: *p* < 0.01) (Figure 2b), validating the participation of GALR2 in the Y1R/GALR2 agonist interaction to stimulate cell proliferation on the ventral hippocampus.

Moreover, the intranasal administration of the Y1R agonist alone induced an increase in the number of PCNA‐positive cells in the Sgz of the ventral hippocampus (Figure 2a,b) compared with the control and M1145 groups Newman–Keuls post hoc test: *p* < 0.05) (Figure 2a,b). However, the intranasal delivery of M1145 alone lacked effects on the numbers of PCNA‐IR profiles (Figure 2b) compared with the control group (Figure 2a–c).

We then sought to identify which cellular types were affected by the intranasal infusion of M1145 and the Y1R agonist. For this, we also quantified the number of PCNA‐labeled cells coexpressing either the DCX‐expressing neuroblasts or GFAP‐expressing quiescent radial stem cells (Figure 2e). The number of PCNA+/DCX+ cells increased after the intranasal infusion of M1145 and the Y1R agonist compared to control group (*t* = 6.063, *df* = 6; *p* < 0.001; Figure 2f). We found that there was no significant change in the number of PCNA+/GFAP+ cells in M1145‐Y1R‐treated animals compared to the control group (*t* = 1.192, *df* = 6; *p* < 0.28). This result indicates that the M1145 and the Y1R agonist stimulate the proliferation of neuroblasts (DCX+), while having no effect on the quiescent radial stem (GFAP+).

### 3.3 Enhanced cell proliferation is related to increased BDNF upon M1145 and Y1R agonist coactivation

**Figure 3:**
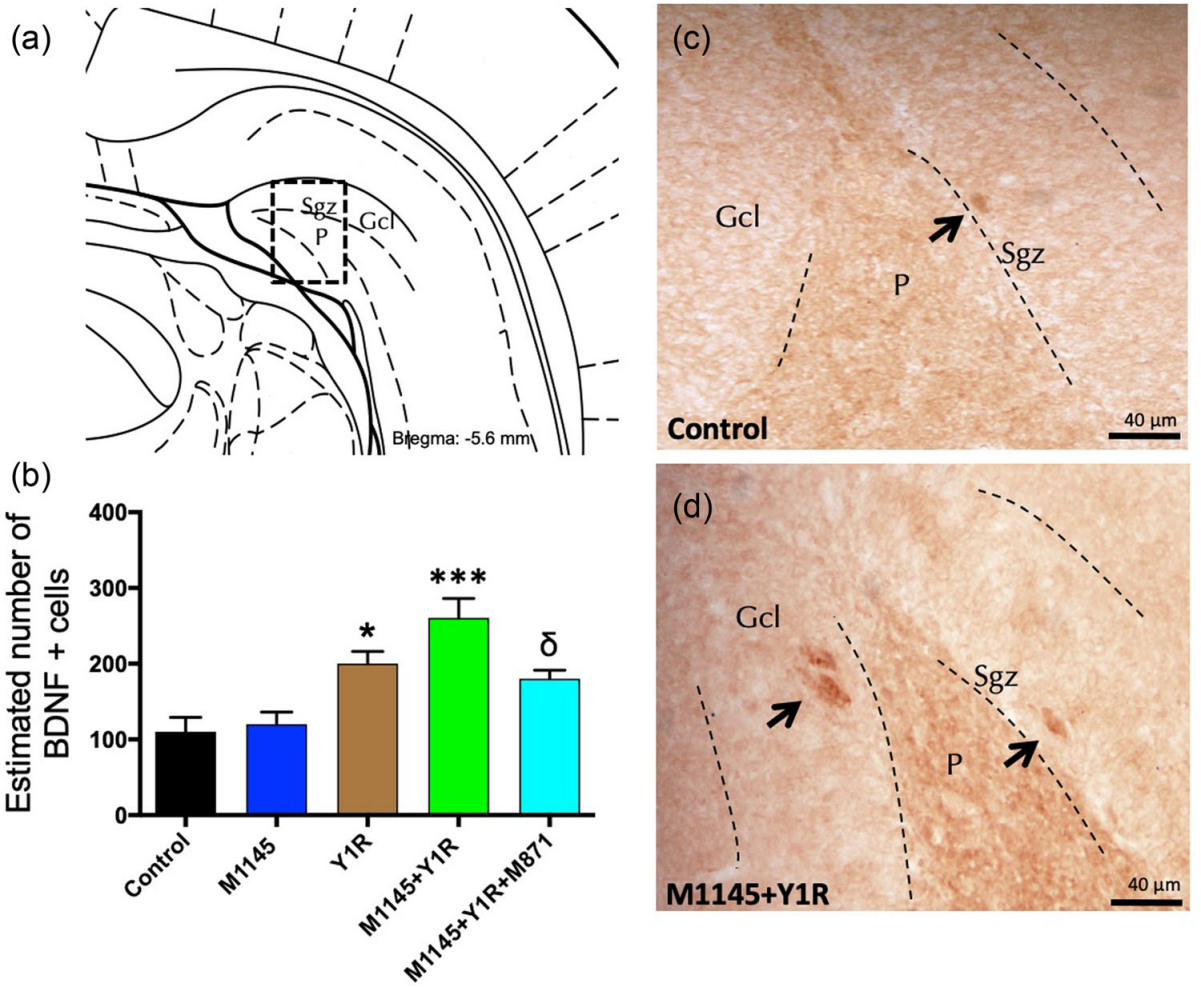
Effects induced by M1145 and Y1R agonist on hippocampal brain‐derived neurotrophic factor immunoreactive (BDNF‐IR) cells of the dentate gyrus (DG) hippocampal region. (a) BDNF‐IR cells were located mainly in the subgranular zone (Sgz) of the dentate gyrus at the border of the granular cell layer (Gcl), some scattered cells were found in the polymorphic layer (P) of the dentate gyrus in the ventral hippocampus (Bregma: –5.6 mm; according to the Paxinos and Watson (2006) stereotaxic atlas). (b) Quantitative analysis of BDNF‐IR cells of the DG. *p < 0.05 versus control, M1145 and M1145 + Y1R; ^δ^ *p* < 0.05 versus M1145 + Y1R; ***p < 0.001 versus control and M1145 according to one‐way ANOVA followed by Newman–Keuls post hoc test (*n* = 4 in each group). The vertical lines from the horizontal line above the bars indicate the Intergroup comparisons. (c, d) Representative microphotographs showing the increase in the BDNF‐positive cells in the DG after M1145 and Y1R agonist coinjection (d) compared with the control group (c). Black arrows point to BDNF‐IR cells. Dashed lines outline the Gcl of the dentate gyrus. ANOVA, analysis of variance; Control, distilled water; M1145, galanin 2 receptor agonist 132 µg; M1145 + Y1R, coadministration of M1145 and Y1R; M1145 + Y1R + M871, coadministration of M1145, Y1R, and GALR2 antagonist M871 132 µg; Y1R, Y1R receptor agonist [Leu^31^–Pro^34^]NPY 132 µg.

To study the cellular mechanism related to the observed effects on cell proliferation, we study the BDNF expression on the ventral hippocampal DG after M1145 and/or Y1R agonist intranasal administration. BDNF‐positive cells were found specifically in the Sgz of the ventral hippocampus, and some scattered cells were observed in the polymorphic layer (P) of the ventral DG (Figure 3a). Stereological quantification of BDNF‐positive cells demonstrated a significant increase after the intranasal coinjection of M1145 and YR1 agonist compared to control (one‐way ANOVA, *F*4, 15 = 11.12, *p* < 0.001, Newman–Keuls post hoc test: *p* < 0.001), M1145 (Newman–Keuls post hoc test: *p* < 0.001) or the YR1 agonist alone (Newman–Keuls post hoc test: *p* < 0.05) (Figure 3a–d).

The intranasal infusion of the Y1R agonist induced a significant increase in the number of BDNF‐positive cells in the ventral DG (Newman Keuls post hoc test: *p* < 0.05) (Figure 3b) compared with the control and the M1145 groups. However, the intranasal infusion of M1145 alone lacked effects on the number of BDNF‐positive cells in the ventral DG. Likewise, to the PCNA‐IR response described above, the presence of the GALR2 antagonist M871 completely blocked the increase induced by the coinjection (Newman–Keuls post hoc test: *p* < 0.05) (Figure 3b), demonstrating the involvement of GALR2 in this interaction.

### 3.4 GALR2 agonist and Y1R agonist interaction enhanced GALR2/Y1R heteroreceptor complexes and neurite length on hippocampal neuronal cells

To study at the receptor level the cellular mechanisms related to the observed in vivo effects we performed in situ PLA on hippocampal neuronal cells. This procedure allowed us to analyze the GALR2/Y1R heteroreceptor complexes formation after M1145 and/or Y1R agonist incubation.

**Figure 4:**
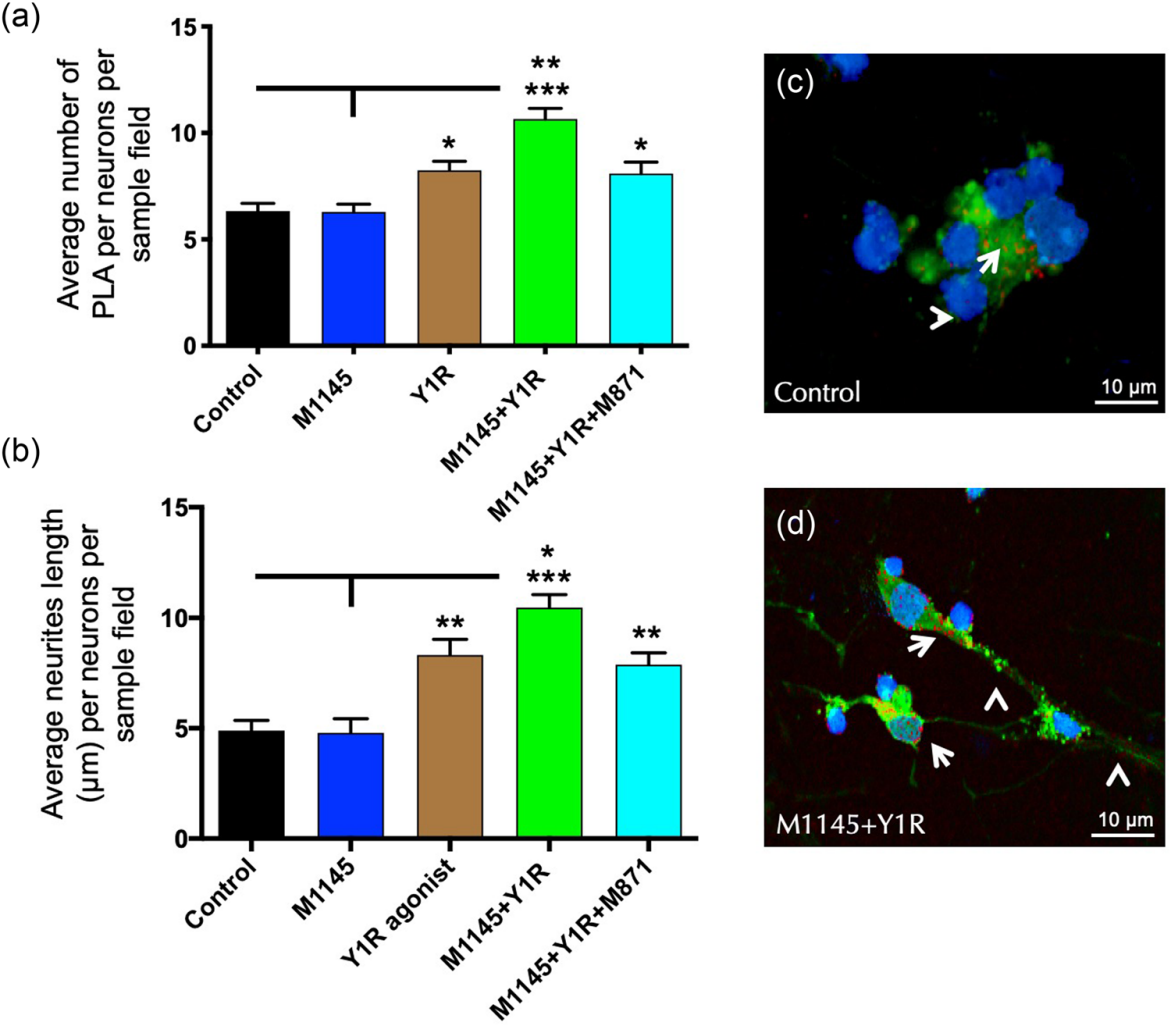
Demonstration of galanin receptor 2 (GALR2)/Y1R heteroreceptor complexes by in situ PLA and morphological changes on hippocampal neuronal cells. (a) The presence of positive PLA signals (red circles) was determined by using the in situ proximity ligation assay (in situ PLA) on hippocampal neuronal cells after treatment with GALR2 agonists (M1145, 100 nM) and Y1R receptor agonist (100 nM), either alone or in combination with or without the GALR2 receptor antagonist (M871, 1 μM). Quantification of PLA signals was performed by measuring red PLA positive blobs per nucleus per sampled field by an experimenter blind to treatment conditions. *p < 0.05 versus control and M1145; **p < 0.01 versus Y1R and M1145 + Y1R + M871; ***p < 0.001 versus control and M1145 according to one‐way ANOVA followed by Newman–Keuls post hoc test. Intergroup comparisons are indicated by the vertical lines from the horizontal line above bars. Data are expressed as mean ± SEM. (b) GALR2 and Y1R agonists modulation of neurites length. The length of neurites per cell was determined after immunofluorescent labeling of neurons and neuronal nuclei (Pan Neuronal Marker (ABN2300)/neuronal nuclei [DAPI]). Quantification is shown in (b), where the data are presented as mean ± SEM. *p < 0.05 versus Y1R and M1145 + Y1R + M871; **p < 0.01 versus control and M1145; ***p < 0.001 versus control and M1145 according to one‐way ANOVA followed by Newman–Keuls post hoc test. (c, d) Representative microphotographs of the significant increase in the density of GALR2/Y1R heteroreceptor complexes (PLA clusters) and neurites length per hippocampal neuronal positive cell after M1145 and Y1R agonist treatment (d) compared with the control group (c). Receptor complexes are shown as red PLA blobs (clusters, indicated by white arrows) found in high densities per hippocampal neuronal cell using confocal laser microscopy. The nuclei are shown in blue by DAPI staining and the cells in green are hippocampal neurons‐positive (Pan Neuronal Marker, ABN2300) using confocal laser microscopy. White arrowheads point to neurite extensions. ANOVA, analysis of variance; Control, culture medium; DAPI, 4′,6‐diamidino‐2‐phenylindole; M1145, galanin 2 receptor agonist 100 nM; M1145 + Y1R, coadministration of M1145 and Y1R; M1145 + Y1R + M871, coadministration of M1145, Y1R, and GALR2 antagonist 1 μM; PLA, proximity ligation assay; Y1R agonist, Y1R receptor agonist [Leu^31^–Pro^34^]NPY 100 nM.

PLA‐positive red clusters were found specifically in the membrane and cytoplasmatic region of hippocampal neuronal cells (Figure 4a–d). Quantification of PLA demonstrated an increase in the density of the PLA‐positive red clusters after Y1R agonist incubation compared to control (one‐way ANOVA, *F*4, 20 = 16.25, *p* < 0.001, Newman–Keuls post hoc test: *p* < 0.05) or M1145 incubation (Newman–Keuls post hoc test: *p* < 0.05) (Figure 4a,c,d). Moreover, upon incubation with M1145 and the Y1R agonist significantly increased the number of PLA‐positive red clusters in the hippocampal neuronal cells (Figure 4a,d) compared to control (Newman–Keuls post hoc test: *p* < 0.001), M1145 alone (Newman–Keuls post hoc test: *p* < 0.001) and Y1R agonist alone (Newman–Keuls post hoc test: *p* < 0.01). Moreover, the specific GALR2 antagonist M871 counteracted this synergic effect (Newman–Keuls post hoc test: *p* < 0.01) (Figure 4a), demonstrating that this interaction was mediated through the coactivation of GALR2 and Y1R.

Due to the changes observed in the density of these complexes upon treatments, we decided to study morphology and structural plasticity changes of hippocampal cultures upon these pharmacological treatments. The data show a significant synergistic increase of mean neurite length upon coactivation of M145 and the Y1R agonist for 24 h compared to the YR1 agonist group alone (one‐way ANOVA, *F*4, 20 = 16.91, *p* < 0.001, Newman–Keuls post hoc test: *p* < 0.05), control (Newman–Keuls post hoc test: *p* < 0.001) and M1145 alone (Newman–Keuls post hoc test: *p* < 0.001) (Figure 4b–d). The Y1R agonist incubated alone increased the neurite length compared to the control and M1145 alone (Newman–Keuls post hoc test: *p* < 0.01) (Figure 4b). Furthermore, the presence of GALR2 antagonist M871 entirely blocks the synergic effects (Newman–Keuls post hoc test: *p* < 0.05) (Figure 4b).

### 3.5 Enhancement of antidepressant‐like response by intranasally‐administered GALR2 and Y1R agonists in the FST

We performed the FST to achieve the functional outcome related to the findings on the ventral hippocampus after the intranasal delivery of GALR2 and Y1R agonists. Rats were pre‐exposed to water for 15 min in the FST and 24 h after the intranasal administration the immobility and swimming parameters were measured during the 5 min test phase to assess signs of depression‐like behavior.

The dose–response curve showed that intranasal infusion of GALR2 agonist lacked effects at 68 and 132 µg in the FST. Regarding the Y1R agonist, the 68 µg dose was ineffective while the 132 µg induced a significant decrease in the immobility time (one‐way ANOVA, *F*4, 25 = 3.79, *p* < 0.05, Supporting Information: Figure 1a) compared to the rest of the groups (Newman–Keuls post hoc test: *p* < 0.05; Supporting Information: Figure 1a). Moreover, an increase in the swimming behavior (one‐way ANOVA, *F*4, 25 = 3.57, *p* < 0.05; Supporting Information: Figure 1b) was observed compared to the rest of the groups (Newman–Keuls post hoc test: *p* < 0.05; Supporting Information: Figure 1b).

**Figure 5:**
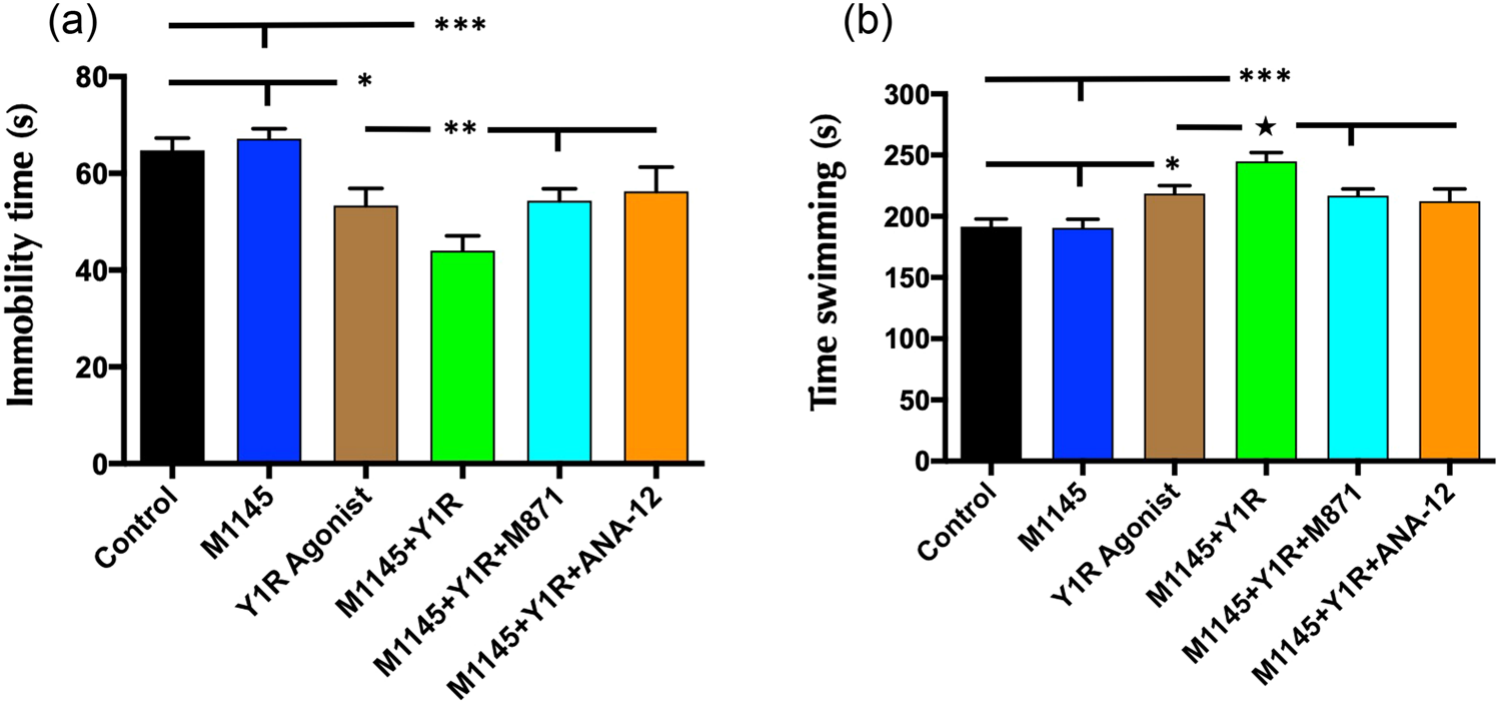
Behavioral actions induced by galanin 2 receptor (GALR2) agonist (M1145) and the neuropeptide Y (NPY) Y1 receptor agonist (Y1R agonist) alone and in combination in the forced swimming test (FST). An antidepressant‐like effect in the FST was observed after M1145 and Y1R agonist in coadministration following a 24 h delay. Furthermore, this effect is counteracted by the GALR2 antagonist M871. Cumulative behavioral duration of immobility (a) and swimming (b) time in the FST. Data represent mean ± SEM. *N* = 6 animals in each group. For (a): *p < 0.05 versus control and M1145; **p < 0.01 versus Y1R agonist, M1145 + Y1R + M871 and M1145 + Y1R + ANA‐12; ***p < 0.001 versus control and M1145. For (b): *p < 0.05 versus control and M1145; ^★^ *p* < 0.05 versus Y1R agonist, M1145 + Y1R + M871 and M1145 + Y1R + ANA‐12; ***p < 0.001 versus control and M1145 according to one‐way ANOVA followed by Newman–Keuls post hoc test. Intergroup comparisons are indicated by the horizontal and vertical lines above bars. ANOVA, analysis of variance; Control, distilled water; in, intranasal; M1145, galanin 2 receptor agonist 132 µg; M1145 + Y1R, coadministration of M1145 and Y1R; M1145 + Y1R + ANA‐12, coadministration of M1145, Y1R, and TrkB antagonist ANA‐12 0.5 mg/kg; M1145 + Y1R + M871, coadministration of M1145, Y1R, and GALR2 antagonist M871 132 µg; Y1R, Y1R receptor agonist [Leu^31^–Pro^34^]NPY 132 µg.

Upon the intranasal coadministration of M1145 and the Y1R, a significant decrease in the immobility time (one‐way ANOVA, *F*5, 30 = 8.96, *p* < 0.001, Figure 5a) was observed compared with control animals (Newman–Keuls post hoc test: *p* < 0.001; Figure 5b), M1145 (Newman–Keuls post hoc test: *p* < 0.001; Figure 5b) and Y1R agonist alone (Newman–Keuls post hoc test: *p* < 0.01; Figure 5b). Moreover, an increase in the swimming behavior (one‐way ANOVA, *F*5, 30 = 10.58, *p* < 0.001; Figure 5b) was observed after the combined treatment compared to the control animals (Newman–Keuls post hoc test: *p* < 0.001; Figure 5b), M1145 (Newman–Keuls post hoc test: *p* < 0.001; Figure 5b) and Y1R agonist alone (Newman–Keuls post hoc test: *p* < 0.05; Figure 1b). GALR2 contribution in this result was confirmed since the addition of the GALR2 antagonist M871 counteracted the synergistic effects on immobility (Newman–Keuls post hoc test: *p* < 0.01; Figure 5b) and swimming (Newman–Keuls post hoc test: *p* < 0.05; Figure 5b) induced by the coadministration of M1145 and the Y1R agonist in the FST. Importantly, we confirmed the BDNF influence in this action since the addition of the TrkB antagonist ANA‐12 blocked the synergistic on immobility (Newman–Keuls post hoc test: *p* < 0.01; Figure 5b) and swimming (Newman–Keuls post hoc test: *p* < 0.05; Figure 5b) induced by the coadministration of M1145 and the Y1R agonist in the FST.

The intranasal infusion of the Y1R agonist decreased the time of immobility compared to the control (Newman–Keuls post hoc test: *p* < 0.05; Figure 5b) and the M1145 (Newman–Keuls post hoc test: *p* < 0.05; Figure 5b) animals; while induced an increase in the swimming behavior compared to the control (Newman–Keuls post hoc test: *p* < 0.05; Figure 5b) and the M1145 (Newman–Keuls post hoc test: *p* < 0.05; Figure 5b) groups. However, the administration of the GalR2 agonist M1145 alone lacked effects on the FST (Figure 5b) compared with the control group.

## 4 DISCUSSION

The present study demonstrates for the first time that GALR2 and Y1R agonists intranasal infusion stimulates adult ventral hippocampal neurogenesis and exerts an antidepressant‐like effect. Intranasal delivery offers an alternative to icv infusion to bypass the blood–brain barrier for the direct delivery of peptides and protein therapeutics rapidly into the CNS, supported by robust evidence in preclinical and clinical trials (Lochhead & Thorne, 2012; Rawal et al., 2022). This method will be crucial as novel therapies continue to be studied in clinical trials and have several benefits, such as fewer side effects than peripheral administration and the comfort of noninvasiveness application (Chapman et al., 2013; Crowe & Hsu, 2022). Accordingly, intranasal esketamine has recently come into the market as an antidepressant, but its use is limited due to potential neurotoxicity, psychocomimetic side effects, potential abuse, and interindividual variability in treatment response (Langmia et al., 2022).

Following intranasal delivery of GALR2 and Y1R agonists we observed an increased cell proliferation in the ventral DG of the hippocampus by using the PCNA. In agreement, we have recently observed the ability of the coagonist intranasal infusion to enhance the cell proliferation in the DG of the dorsal hippocampus at 24 h (Borroto‐Escuela et al., 2022). Previous work showed that icv infusion of GAL and the Y1R agonist‐induced proliferation by using 5‐bromo‐2‐deoxyuridine on the ventral hippocampus (Borroto‐Escuela et al., 2021). Importantly, the strength of the present study is represented by the intranasal delivery of both, GALR2 and Y1R specific agonists, offering a noninvasive route to icv injection. Interestingly, enhanced resilience in a model of depression was recently demonstrated by genetically boosting neurogenesis in the ventral hippocampal DG (Planchez et al., 2021). Furthermore, the molecule P7C3 increased cell proliferation in the hippocampal DG associated with antidepressant effects in rodents and primates (Bauman et al., 2018; Walker et al., 2015). Moreover, intranasal administration of the Y1R agonist alone increased cell proliferation in the ventral DG in this work. However, we observed that the Y1R agonist lacks effects on cell proliferation in the dorsal DG (Borroto‐Escuela et al., 2022; Mirchandani‐Duque et al., 2022). These results confirm the functional differences described between ventral and dorsal parts and a differential role for NPY in these subregions of the hippocampal formation (Baptista & Andrade, 2018; Lee et al., 2017). Regarding the intranasal administration of the GALR2 agonist alone, we observed no effects on ventral hippocampal cell proliferation. Previously, it was reported that GalR2/3 mediated the proliferative and trophic effects of GAL (Abbosh et al., 2011), indicating in subsequent studies a role for GALR3 (Khan et al., 2017). However, these studies were performed in vitro conditions, exhibiting significant differences in systems in vivo. We then sought to determine whether a specific cell subpopulation was more affected by M145 and Y1R agonist coadministration. For this, out of the overall PCNA+ cells quantified, we identified and quantified the number of cells coexpressing the neuroblast marker DCX (PCNA+/DCX+ cells) or the quiescent radial stem cells marker (PCNA+/GFAP+ cells). Our data indicate that M1145 and the Y1R agonist stimulate the proliferation of neuroblasts while having no effect on quiescent neural progenitors and astrocytes. In agreement, previous reports show that NPY promotes the proliferation of amplifying neural progenitors and neuroblasts (Decressac et al., 2011; Howell et al., 2005, 2003).

Dysregulation in subventricular zone (SVZ) neurogenesis is described in many neurodegenerative diseases. AD and Parkinson's disease showed reduced stem cell proliferation, while stroke and Huntington's disease induce an enhancement of SVZ neurogenesis to migrate into damaged areas (Curtis et al., 2007). Intriguingly, NPY was described to induce a proneurogenic role through Y1R on DCX‐positive neuroblasts and to exert a direct role in cell migration (Decressac et al., 2009; Thiriet et al., 2011). Further research is required to explore the potential role of GALR2 and Y1R agonists intranasal administration in cell replacement‐based strategies in neurodegenerative diseases affecting SVZ neurogenesis.

At the cellular level, these effects on hippocampal cell proliferation after GALR2 and Y1R agonists intranasally coadministered seems to be mediated by increased BDNF expression on the ventral hippocampal DG. BDNF, a member of neurotrophins, has a pivotal role in increasing neurogenesis through changes in proliferation and cell survival (Miranda et al., 2019). Recent evidence showed that physical exercise protects the brain from depressive symptoms through increasing hippocampal neurogenesis combined with BDNF (Murawska‐Ciałowicz et al., 2021). Thus, therapeutics that promote the close correlation between dentate neurogenesis and BDNF, as seen under the GALR2 and Y1R agonist combination, maybe the key to preventing or curing depression. In this regard, our data are consistent with previous evidence on the BDNF‐related neuroprotective effect of NPY in models of neurodegeneration (Corvino et al., 2012; Croce et al., 2011).

These cellular effects induced by GALR2 and the Y1R agonist were achieved in hippocampal neuronal cells by studying GALR2/Y1R heteroreceptor complexes upon agonist coactivation of both receptor protomers. We observed an increase of GALR2/Y1R heteroreceptor complexes by using in situ PLA upon combined coincubation with GALR2 and Y1R agonists. We have previously reported the presence of GALR2/Y1R heteroreceptor complexes in HEK cells and several limbic brain regions, including the amygdala and the dorsal hippocampus (Borroto‐Escuela et al., 2021; Narváez et al., 2016, 2018, 2015. Additionally, we confirmed how 5HT1A–FGFR1 heteroreceptor complexes significantly stimulated hippocampal plasticity linked to antidepressant‐like actions (Borroto‐Escuela et al., 2012; Narváez et al., 2020). Besides, GALR2 and Y1R agonist coincubation promoted an increment of the length of the neurite in hippocampal neuronal cells at 24 h, where BDNF might be a common mechanism in our in vivo and in vitro experiments. In agreement, it was shown that BDNF exerted a promoting effect on dendritic outgrowth in primary hippocampal cultures and the hippocampus (H. I. Kim et al., 2022; Park et al., 2016).

The functional outcome was certified by demonstrating the enhancement of the antidepressant‐like response after the intranasal delivery of GALR2 and Y1R agonists on the FST at 24 h. In previous reports, the intranasal infusion of the Y1 agonist (Serova et al., 2017) and NPY (Nahvi et al., 2021) in rats or NPY in humans (Mathé et al., 2020) induced antidepressant effects for at least 24 h. In agreement, the N‐methyl‐d‐aspartate receptor antagonist, Ketamine, or the group II metabotropic glutamate (mGlu2/3) receptor antagonist, LY341495 were shown to exhibit antidepressant‐like effects after a single injection in the FST in rats at 24 h (Gigliucci et al., 2013; Koike & Chaki, 2014). We found that the intranasal administration of the GALR2 agonist alone lacks antidepressant‐like effects at 24 h. We may speculate that subchronic or chronic intranasal treatments with GALR2 and Y1R agonists would be required in pathological models of depression or to achieve long‐lasting effects. Species–specific differences between rats and mice in antidepressant responses have been reported (Polis et al., 2019). Thus, previous studies indicate that the intranasal infusion of spexin‐based GALR2 agonist‐induced antidepressant‐like effects was performed on mice for 2–3 h (Yun et al., 2019). Recently, intranasal delivery in rats of M39b, a stabilized GALR2 agonist, has been shown as a promising candidate for future applications (Kuipers et al., 2021). Moreover, we found that the GALR2 antagonist M871 counteracted the enhanced response observed, as previously reported after intranasal GALR2 and Y1R agonists (Borroto‐Escuela et al., 2022). In this previous work, we confirmed that the behavioral effects were independent of the motor activity since GALR2, Y1R agonist, or their intranasal coadministration did not show locomotor alterations. In agreement with our findings, the ventral hippocampus was involved in the antidepressant effects of NPY in posttraumatic stress disorder (Sabban & Serova, 2018). Accordingly, we may speculate that the molecular mechanisms underlying the antidepressant‐enhancing actions of the Y1R and GALR2 agonists at 24 h might be mediated by enhancing the signaling of these two protomers in the Y1R–GALR2 heterocomplexes in the neurogenic zone of the ventral hippocampus. Moreover, our data support the contribution of BDNF in this mechanism, as observed in vivo and in vitro experiments. Besides, the TrkB antagonist ANA‐12 was shown to counteract the antidepressant effects of ketamine at 24 h (Ribeiro et al., 2020). Interestingly, running was shown to enhance BDNF signaling and neuronal proliferation in the ventral hippocampus related to antidepressant effects (Murawska‐Ciałowicz et al., 2021).

Taken together, the intranasal infusion of Y1R and GALR2 agonists may promote cell proliferation in the ventral hippocampal DG and the induction of the BDNF neurotrophic factor. These effects may be mediated by Y1R–GALR2 heteroreceptor complexes to mediate increased neurites outgrowth observed on neuronal hippocampal cells. Accordingly, these cellular effects may be linked to the enhanced antidepressant effects observed. In this way, a reorganization of the signaling in this Y1R–GALR2 heteroreceptor complex, including the homoreceptor complex associated with altered hetero/homosignaling might mediate the increased antidepressant actions. Our data may suggest the development of new heterobivalent o multitargeting agonist pharmacophores acting on Y1R–GALR2 heterocomplexes in the ventral hippocampus for the novel therapy of MDD or depressive‐affecting diseases. These new therapy approaches could be designed in future clinical trials using intranasally delivered Y1R and GALR2 agonists.

## CONFLICT OF INTEREST

The authors declare no conflict of interest.

## Supporting information

Supporting information.
