# Supplementary material for: GALR2 and Y1R agonists intranasal infusion enhanced adult ventral hippocampal neurogenesis and antidepressant‐like effects involving BDNF actions
Source: J Cell Physiol. 2023 Jan 4;238(2):459–74. doi: 10.1002/jcp.30944 (PMC10952952; doi:10.1002/jcp.30944)
Supplement: Supplementary file 1 — Supporting information. [file JCP-238-459-s001.docx]

***Supplementary Material***

**Antidepressant augmentation upon intranasal Galanin and Neuropeptide Y agonists enhanced ventral hippocampal proliferative and neurotrophic actions**

**Jose Erik Alvarez-Contino ^1^, Estela Díaz-Sánchez ^1,2^, Marina Mirchandani-Duque ^1^, Jose Andrés Sánchez-Pérez ^2^, Miguel A. Barbancho ^1^, Alexander López-Salas ^1^, Natalia García-Casares ^1^, Kjell Fuxe ^3^, Dasiel O. Borroto-Escuela ^1,3,4,^* and Manuel Narváez ^1,3,5^***

1 Instituto de Investigación Biomédica de Málaga, Facultad de Medicina, Universidad de Málaga,

Malaga, 29071, Spain; marina.md97@uma.es (M.M.-D.); mabarbancho@uma.es (M.A.B.); 0611059553@uma.es (A.L.-S.); 0611059544@uma.es (J.E.A.-C.); nagcasares@uma.es (N.G.-C.); estela_dra@uma.es (E.D.-S.); dasiel@uma.es (D.O. B-E)

2 Laboratorio NeuronLab. Vithas Málaga. Grupo Hospitalario Vithas. Málaga. Spain

3 Instituto de Investigación Biomédica de Málaga, Unit of Psychiatry, Hospital Universitario Virgen de la Victoria. sanchezperezjoseandres@gmail.com

4 Department of Neuroscience, Karolinska Institute, Stockholm, 17177, Sweden; kjell.fuxe@ki.se

5 Department of Biomolecular Science, Section of Physiology, University of Urbino, Urbino, 61029, Italy

* These authors contribute equally to this work

Correspondence: mnarvaez@uma.es (M.N.)

**Intranasal administration of peptides**

Galanin receptor 2 agonist (M1145), Y1R receptor agonist [Leu^31^, Pro^34^]NPY, GALR2 Antagonist M871 (Tocris Bioscience, Bristol, UK) were freshly dissolved in 20 μl distilled water. Each rat received 10 μl of them into each nostril with pipetteman and disposable plastic tip (1 mm in diameter) inserted no deeper than 1–1.5 mm into the nostril under light isoflurane anesthesia. Following the infusion, the head of the animal was held in a tilted back position for approximately 15 s to prevent loss of solution from the nares.

**Counting Procedure**

c-Fos, PCNA and BDNF-labeled cells were counted with an Olympus BX51 microscope, Olympus, Denmark interfaced with a computer and a colour JVC digital video camera. For stereological analysis, sampling of positive cells was performed throughout the dentate gyrus of the ventral hippocampus in the rostrocaudal dimension using the optical fractionator. This method combines the optical dissector with a fractionator sampling scheme to exclude volume divergences (Gundersen et al., 1988). Counterstaining with phase contrast allowed delineation of different areas in each section (Paxinos and Watson, 2006). Numbers of positive cells were quantified in at least five representative 150 μm, evenly spaced sections per animal (4 rats per group). A random set of sampling frames with a known area (α frame) was generated for each section using the C.A.S.T. Grid (Olympus; Albertslund, Denmark). After the objects were counted (ΣQ-) the total number of positive cells were estimated as: N = ΣQ- x fs x fa x fh (Gundersen et al., 1988), where fs is the numerical fraction of the section used, fa is the areal fraction and fh is the linear fraction of section thickness. The quantification was limited to the granular cell layer and subgranular zone. Subgranular region was outlined as a band-limited by three nuclei down from the edge between the granular cell layer and the hilar region, and cells located more than two cells away from the subgranular zone were excluded. The coefficient of error (CE) for each estimation and animal ranged from 0.05 to 0.1. The total CE of each group ranged from 0.07 to 0.08. Counting of labelled cells was set starting at 5 μm below the surface and focusing through the 20 μm section optical plane, and the number of counting frames used was 90-110 per animal. We have used this stereological procedure is previous studies (Narvaez et al., 2016;Narvaez et al., 2018; Borroto-escuela et al., 2022; Mirchandani-duque et al., 2022).

**Supplementary Figure 1**


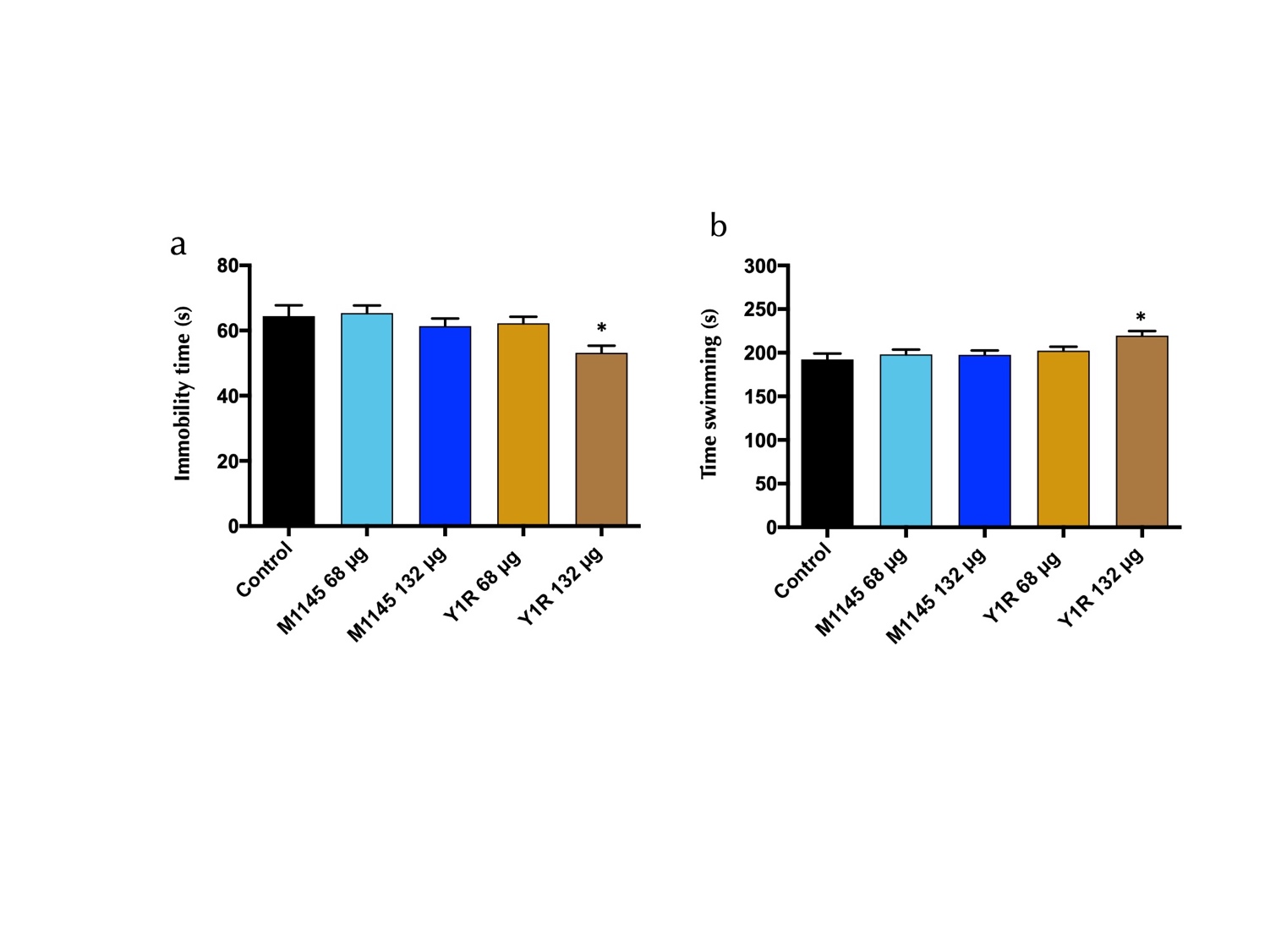


**Supplementary Figure 1.** Dose-response and behavioral actions induced by Galanin 2 receptor agonist (M1145) and the Neuropeptide Y (NPY) Y1 receptor agonist (Y1R agonist) alone at different doses in the forced swimming test (FST). An antidepressant-like effect in the FST was observed after Y1R agonist intranasal at 132 µg following a 24 hours delay. Cumulative behavioral duration of Immobility **(a)** and swimming **(b)** time in the FST. Data represent mean ± SEM. N=6 animals in each group. *P <0.05 vs the rest of the groups according to one-way ANOVA followed by Newman-Keuls post-hoc test. Abbreviations: Control= Distilled water; M1145 68 µg = Galanin 2 receptor agonist 68 µg; M1145 132 µg = Galanin 2 receptor agonist 132 µg; Y1R 68 µg = Y1R receptor agonist [Leu^31^-Pro^34^]NPY 68 µg; Y1R 132 µg = Y1R receptor agonist [Leu^31^-Pro^34^]NPY 132 µg.

**References**

Borroto-Escuela, D. O., Fores, R., Pita, M., Barbancho, M. A., Zamorano-Gonzalez, P., Casares, N. G., . . . Narvaez, M. (2022). Intranasal Delivery of Galanin 2 and Neuropeptide Y1 Agonists Enhanced Spatial Memory Performance and Neuronal Precursor Cells Proliferation in the Dorsal Hippocampus in Rats. Front Pharmacol, 13, 820210. doi:10.3389/fphar.2022.820210

Gundersen, H.J., Bagger, P., Bendtsen, T.F., Evans, S.M., Korbo, L., Marcussen, N., Moller, A., Nielsen, K., Nyengaard, J.R., Pakkenberg, B., and Et Al. (1988). The new stereological tools: disector, fractionator, nucleator and point sampled intercepts and their use in pathological research and diagnosis. APMIS 96, 857-881.

Mirchandani-Duque, M., Barbancho, M. A., Lopez-Salas, A., Alvarez-Contino, J. E., Garcia-Casares, N., Fuxe, K., . . . Narvaez, M. (2022). Galanin and Neuropeptide Y Interaction Enhances Proliferation of Granule Precursor Cells and Expression of Neuroprotective Factors in the Rat Hippocampus with Consequent Augmented Spatial Memory. Biomedicines, 10(6). doi:10.3390/biomedicines10061297

Narvaez, M., et al. (2015). "Galanin receptor 2-neuropeptide Y Y1 receptor interactions in the amygdala lead to increased anxiolytic actions." Brain Struct Funct 220(4): 2289-2301.

Narvaez, M., et al. (2016). "Galanin receptor 2-neuropeptide Y Y1 receptor interactions in the dentate gyrus are related with antidepressant-like effects." Brain Struct Funct 221(8): 4129-4139

Narvaez, M., et al. (2018). "A Novel Integrative Mechanism in Anxiolytic Behavior Induced by Galanin 2/Neuropeptide Y Y1 Receptor Interactions on Medial Paracapsular Intercalated Amygdala in Rats." Front Cell Neurosci 12: 119

Paxinos, G., and Watson, C. (2006). *The rat brain in stereotaxic coordinates: hard cover edition.* Elsevier.

Serova, L.; Mulhall, H.; Sabban, E. NPY1 Receptor Agonist Modulates Development of Depressive-Like Behavior and Gene Expression in Hypothalamus in SPS Rodent PTSD Model. *Front Neurosci* **2017**, *11*, 203, doi:10.3389/fnins.2017.00203.

Serova, L.I.; Hansson, E.; Sabban, E.L. Effect of intranasal administration of neuropeptide Y and single prolonged stress on food consumption and body weight in male rats. *Neuropeptides* **2020**, *82*, 102060, doi:10.1016/j.npep.2020.102060.
